# Is topiramate effective for migraine prevention in patients less than 18 years of age? A meta-analysis of randomized controlled trials

**DOI:** 10.1186/s10194-017-0776-4

**Published:** 2017-07-18

**Authors:** Kai Le, Dafan Yu, Jiamin Wang, Abdoulaye Idriss Ali, Yijing Guo

**Affiliations:** Department of Neurology, Affiliated ZhongDa Hospital, School of Medicine, Southeast University, Nanjing, Jiangsu 210009 People’s Republic of China

**Keywords:** Topiramate, Pediatric, Adolescent, Children, Migraine, Prevention

## Abstract

**Background:**

Mainly based on evidence of success in adults, various medications are commonly used to prevent pediatric migraines. Topiramate has been approved for migraine prevention in children as young as 12 years of age. In this meta-analysis, we aimed to assess the currently published data pertaining to the efficacy of topiramate for migraine prevention in patients less than 18 years of age.

**Methods:**

We searched PubMed/Medline, Embase and the Cochrane Library (from inception to April 2017) for randomized controlled trials (RCTs) published in English. Two independent investigators performed data extraction and quality evaluation using the Cochrane Collaboration’s tool. The data extracted were analyzed by Review Manager 5.3 software.

**Results:**

A total of four RCTs matching the inclusion criteria were included, with an aggregate of 465 patients. Of these patients, 329 were included in the topiramate group, and 136 were included in the placebo group. This meta-analysis revealed that compared with placebo, topiramate failed to decrease the number of patients experiencing a ≥ 50% relative reduction in headache frequency (*n* = 465, RR = 1.26, 95% CI = [0.94,1.67], Z = 1.55, *P* = 0.12) or the number of headache days (*n* = 465, MD = −0.77, 95% CI = [−2.31,0.76], Z = 0.99, *P* = 0.32) but did reduce PedMIDAS scores (*n* = 205, MD = −9.02, 95% CI = [−17.34, −0.70], Z = 2.13, *P* = 0.03). Higher rates of side effects and adverse events in the topiramate group than in the placebo group were observed in the included trials.

**Conclusions:**

Topiramate may not achieve a more effective clinical trial endpoint than placebo in the prevention of migraines in patients less than 18 years of age, and topiramate may lead to more side effects or adverse events in the included patients.

## Background

Migraine is the most common cause of headache in pediatric neurology outpatient clinics, and it has been recognized as one of the most prevalent neurological disorder in children and adolescents worldwide, affecting 5–10% of the pediatric population in multiple areas of life. Because patients miss school and social activities, migraines can impair the development of friendships that are vital to social development and self-esteem and may destroy family harmony [1, 2]. The mean age of onset of migraine is 7.2 years in boys and 10.9 years in girls [3], and the prevalence of migraine increases with age, as demonstrated by clinical studies. The diagnostic criteria for migraine headaches have developed over time; modern migraine classification includes frequency as a criterion, with episodic headaches occurring up to 14 days per month, and chronic migraine is defined as the persistence of headache without aura for at least 15 days per month and for at least 3 consecutive months without medication overuse (ICHD-II) [4]. Because of the diversity of symptoms, the diagnosis of migraines in children and adolescents needs to be refined even further. Due to the harm caused by migraines, reducing the number of migraine attacks to the greatest extent possible should be a priority.

At present, a variety of prophylactic therapy options are available to reduce the frequency or severity of headaches [5]. Topiramate is an antiepileptic drug with positive efficacy and safety for older children and adults with epilepsy [6], and it has been approved for migraine prevention in adults in Europe since 2003 and in the United States since 2004 [7]. The exact mechanism of topiramate in the treatment of migraine is unknown, although it may be associated with the influence of topiramate on pain transmission in the trigeminocervical complex and the third-order neurons in the ventroposteromedial thalamus [8]. Several case series and open-label trials [9–13] have shown that topiramate served as a preventive treatment for pediatric migraines, while the research of Scott W [14] indicated that there were no significant differences between topiramate and placebo in the prevention of pediatric migraine. Hence, in the present study, we performed a meta-analysis of randomized controlled trials (RCTs) to evaluate the efficacy of topiramate for the prevention of migraine in patients less than 18 years of age.

## Methods

### Protocol registration

The protocol registration number was CRD42017062287 (http://www.crd.york.ac.uk/Prospero).

### Data sources and search strategies

We searched using the following databases: PubMed/Medline, Embase and the Cochrane Library (inception to April 2017) to retrieve the RCTs of topiramate in migraine prevention for patients less than 18 years of age. The following search terms were used in combination: (“topiramate” OR “topamax”) AND (“migraine disorders” OR “migraine” OR “migraineur” OR “migraineurs” OR “migrain” OR “sick headache”) AND (“pediatric” OR “adolescent” OR “adolescence” OR “child” OR “children” OR “childhood” OR “teen” OR “youth”). The references of eligible studies, relevant systematic reviews, and meta-analysis were also manually retrieved. The publication language was restricted to English.

### Study selection

The automatically retrieved studies were evaluated by two independent investigators and included in the meta-analysis based on the criteria presented below. The reviewers resolved any differences by consensus. The investigators selected the retrieved studies that matched the inclusion and exclusion criteria.

#### Inclusion criteria

Studies were included in the meta-analysis if they fulfilled the following criteria: (1) the study was a trial comparing topiramate with placebo in migraine patients, (2) the study had similar diagnostic criteria for migraine or definition of migraine, (3) the age of the participants was less than 18 years, (4) the study was a clinical RCT, (5) the intent-to-treat population numbers in the topiramate and placebo groups were provided, and (6) the number of participants showing ≥50% reduction in headache frequency, baseline and follow-up data of headache days or PedMIDAS scores were available.

#### Exclusion criteria

Studies were excluded according to the following exclusion criteria: (1) the study was not a RCT but a review, case report, letter, editorial or other type of publication not describing original research, (2) the full text was not available, (3) the study did not afford extractable outcomes, (4) the control group of the trial did not contain placebo (for example, the trial only used propranolol or sodium valproate as a control), and (5) the trial involved adults and children, but the characteristics or outcomes of the pediatric subgroup were unavailable or unextractable.

### Risk of bias in individual studies

The methodological quality of RCTs was evaluated according to the risk of bias tool described in the Cochrane Handbook for Systematic Reviews of Interventions [15]. Seven quality elements that contain random sequence generation, allocation concealment, blinding of participants and personnel, blinding of outcome assessment, incomplete outcome data, selective reporting and baseline balance bias were assessed. Study selection, data extraction and risk bias assessment were conducted by two researchers (Kai Le and Dafan Yu) independently; in case of discrepancies consensus was reached by discussion with a third party (Yijing Guo).

### Data extraction

Our primary outcome was a relative reduction in the number of headache days of 50% or more in the comparison of the 28-day baseline period with the last 28 days. Secondary outcomes included headache days and PedMIDAS scores. The PedMIDAS score, which is used to ascertain a change in headache-related disability [16] between the beginning and the end of the trial and the decrease in the number of headache days from the 28-day baseline period to the final 28-day period of treatment were recorded. The main information, including the numbers of participants in the topiramate and placebo groups, the diagnostic tool, the dose and duration of topiramate, the numbers of patients experiencing a ≥ 50% relative reduction in headache frequency, the mean headache days per 28-day period and the mean PedMIDAS score in both groups, was extracted. Additional information was also abstracted, such as publication year, first author, age and sex. Side effects and adverse events after drug administration were also recorded if they occurred. The two investigators (Kai Le and Dafan Yu) extracted all the data independently. If there was any disagreement between the two reviewers, they resolved it by discussion and consensus, with a third party participating if necessary.

### Statistical analysis

The meta-analysis was conducted using Review Manager 5.3 software (Cochrane Collaboration, Copenhagen, Denmark). Continuous data are presented as the mean difference (MD) with a 95% confidence interval (CI) and inverse variance (IV). Dichotomous outcomes were analyzed by pooled risk ratio (RR) with a 95% CI to present effect estimate and Mantel-Haenszel test. The heterogeneity among eligible trials was quantified using a chi-squared-based *Q*-statistic test (*P* < 0.1, suggesting the existence of heterogeneity). An *I* ^*2*^ statistic was alsoused to quantify the inconsistency across studies, with *I* ^*2*^ > 50% considered statistically significant. When there was no statistically significant heterogeneity, we used a fixed-effects model for pooling the results; otherwise, a random-effects model was used. A 2-sided *P* value <0.05 was taken to indicate statistical significance for 1 comparison group over the other. The results of the meta-analysis were visualized using forest plots. Visual inspection of funnel plots was used to assess possible publication bias if more than 10 trials were identified that reported on the same outcome [17].

## Results

### Search results

**Fig. 1:**
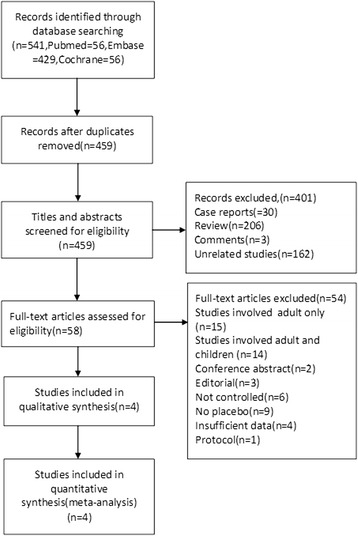
Flow diagram of the study selection process

A total of 541 articles were identified from among 56 listed in PubMed/Medline, 429 in Embase and 56 in the Cochrane Library. After excluding 82 duplicates, 459 potentially eligible articles were selected. Of these articles, 401 were excluded through titles and abstracts, leaving 58 articles for further evaluation. The reasons for exclusion during full-text review were “studies involved adults only” (*n* = 15), “studies included adults and children” (*n* = 14), “conference abstract” (*n* = 2), “editorial” (*n* = 3), “not controlled” (*n* = 6), “no placebo” (*n* = 9), “insufficient data” (*n* = 4) and “protocol” (*n* = 1). Finally, 4 prospective RCTs [14, 18–20] were included in our meta-analysis. The research process is shown in Fig. 1.

### Characteristics of the included RCTs

The 4 included studies were published between 2005 and 2017. Study sample sizes ranged from 42 to 163, with a total of 465 randomized patients, including 329 patients in the topiramate group and 136 in the placebo group, and the age of the participants varied from 8 to 17 years old. To diagnose a migraine, one trial [20] employed the International Headache Society (IHS) diagnostic criteria for pediatric migraine and 3 trials [14, 18, 19] used the International Classification of Headache Disorders, 2nd Edition (ICHD-II) [21]. One trial [18] used 2 doses of topiramate versus placebo. Another trial [14] used amitriptyline and topiramate as the two treatment arms, and we extracted the results of topiramate versus placebo. The duration of the included trials consisted of titration and maintenance periods: 2 trials [18, 19] lasted 16 weeks, one trial [20] lasted 20 weeks, and one trial [14] lasted 24 weeks. The detailed characteristics of the studies included in the meta-analysis are listed in Table 1.

**Table 1:** Characteristics of Studies Included in the Meta-Analysis

First authors, year	Diagnostic tool	Topiramate						Placebo		
		N	Age (years),mean ± SD	Gender (male:female,%)	Dose& Duration		Side effects/Adverse events	N	Age (years),mean ± SD	Gender (male: female,%)
					Titration	Maintenance				
Paul Winner,2005	International Headache Society (IHS) diagnostic criteria for pediatric migraine	108	11.3 ± 2.5	50.9:49.1	8-week: Week 1 = 15 mg/d Week 2 = 30 mg/d Week 3 = 50 mg/d (dose increased to 2–3 mg/kg/d)	12-week: 2–3 mg/kg/d	upper respiratory tract infection, anorexia, weight decrease, gastroenteritis, paresthesia, somnolence	49	10.7 ± 2.6	53.1:46.9
C. V. S. Lakshmi,2007	International Classification of Headache Disorders, 2nd Edition (ICHD-II)	21	10.95 ± 1.53	85.7:14.3	1-month: 25-mg incremented to 100 mg/d	3-month: 100 mg/d	weight loss, lack of concentration in school, parasthesia, sedation, loss of appetite, pain in abdomen	21	10.14 ± 1.35	52.4:47.6
Donald Lewis,2009	International Classification of Headache Disorders, 2nd Edition (ICHD-II)	70^†^	14.2 ± 1.54	40:60	4-week: 25-mg incremented to 50 or 100 mg/d^†^	12-week: 50 or 100 mg/d	upper respiratory tract infection, paresthesia, abdominal pain, anorexia, injury, rhinitis, coughing, viral infection, pharyngitis, fatigue, nausea, dizziness, taste perversion, insomnia, back pain, conjunctivitis, sinusitis, asthma, pneumonia, fever, allergy, vomiting, nervousness, somnolence, abnormal vision, eye pain	33	14.4 ± 1.7	36:64
Scott W.Powers,2017^a^	International Classification of Headache Disorders, 2nd Edition (ICHD-II)	145	14.2 ± 2.5	30:70	8-week: 2 mg/Kg.d, dose escalation occurred every 2 weeks	16-week: average 1.93 ± 0.40 mg mg/Kg.d	aphasia, cognitive disorder, dizziness, memory impairment, paresthesia, general: fatigue, dry mouth, intussusception, streptococcal pharyngitis, upper respiratory tract infection, altered mood, suicide attempt, investigations: decreased weight, contusion, traumatic liver injury, respiratory: bronchospasm	33	14.2 ± 2.2	32:68

*SD standard deviations, N Total number in group*

^a^The analysis population included patients who ended trial early

†35 subjects treated with topiramate at 50 mg/day, 35 subjects treated with topiramate at 100 mg/day

### Risk of bias of the included trials

**Fig. 2:**
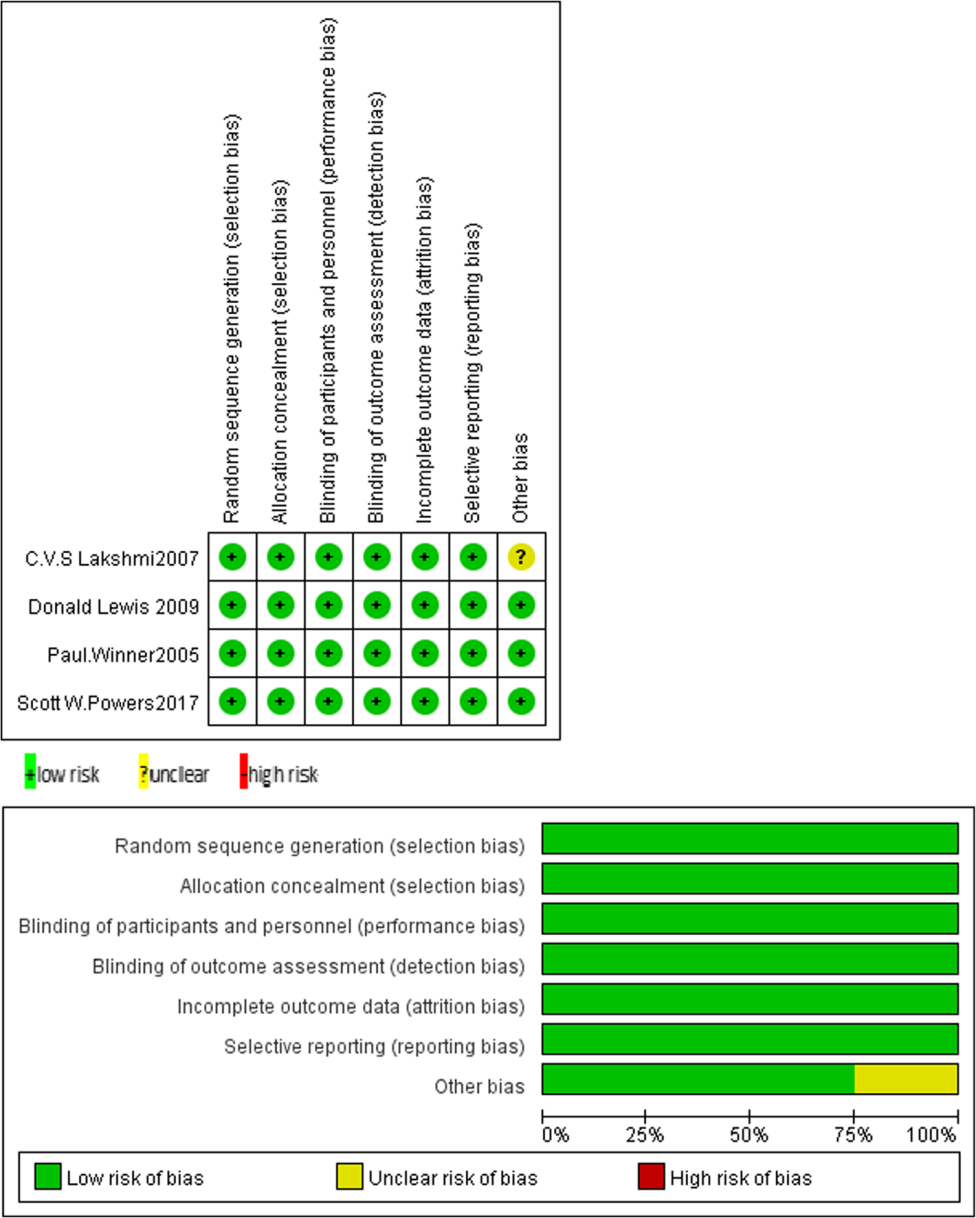
Risk of bias summary. Presentation of the risk of bias summary of the review authors

All RCTs described the procedure of randomization and blinded participants and researchers, and all trials reported allocation concealment and blinding of outcome assessment. All outcome data were complete. Detailed information is shown in Fig. 2.

### Meta-analysis

#### Primary outcome

**Fig. 3:**
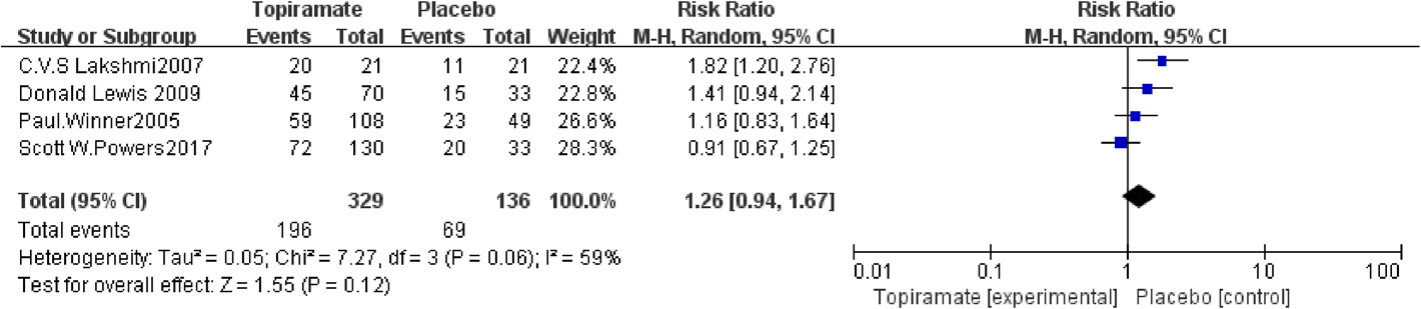
Forest plot of comparison:≥50% Relative reduction in headache frequency of topiramate versus placebo

As shown in Table 2, all 4 included trials investigated the effects of topiramate on migraine prevention via the numbers of patients experiencing a ≥ 50% relative reduction in headache frequency. The results of our meta-analysis show that, there were no significant differences between the topiramate and placebo groups in terms of the numbers of patients experiencing a ≥ 50% relative reduction in headache frequency (*n* = 465, RR = 1.26, 95% CI = [0.94, 1.67], *Z* = 1.55, *P* = 0.12) (Fig. 3). The evidence collected in our meta-analysis shows heterogeneity (*I* ^*2*^ = 59%). Analysis was performed by a random-effects model. The *z*-test result for overall effects showed no statistical significance (*P* = 0.12).

**Table 2:** Trial outcomes of Studies Included in the Meta-Analysis

First authors, year	Headache days per 28-day period (d), mean ± SD		PedMIDAS score, mean ± SD		≥ 50% Relative reduction in headache frequency,n(%)	
	Topiramate	Placebo	Topiramate	Placebo	Topiramate	Placebo
Paul Winner,2005	3.1 ± 3	2.4 ± 2.8	NA	NA	59(54.63)	23(46.94)
C. V. S. Lakshmi,2007	4.27 ± 1.95	7.48 ± 5.94	10.2 ± 6.39	23.7 ± 11.9	20(95.24)	11(55)
Donald Lewis,2009	2.4 ± 3.11	3.5 ± 3.47	NA	NA	45(64.29)	15(45.45)
Scott W.Powers,2017	4.6 ± 5.3	5.2 ± 6.5	14.4 ± 17.3	19.4 ± 20.8	72(55.38)	20(60.61)

*SD* standard deviations*, NA* not available

#### Secondary outcomes

**Fig. 4:**
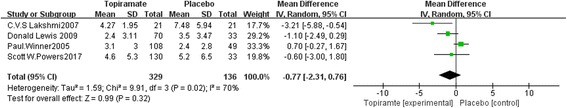
Forest plot of comparison: headache days per 28-day period of topiramate versus placebo

**Fig. 5:**
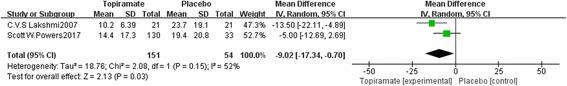
Forest plot of comparison: PedMIDAS score of topiramate versus placebo

All 4 trials included in our meta-analysis reported mean headache days from the 28-day baseline period to the final 28-day period of treatment, and 2 trials [14, 19] presented mean PedMIDAS scores (Table 2). We found no significant difference in mean headache days between the topiramate and placebo groups (*n* = 465, MD = −0.77, 95% CI = [−2.31, 0.76], *Z* = 0.99, *P* = 0.32) (Fig. 4). The evidence collected in our meta-analysis shows considerable heterogeneity (*I* ^*2*^ = 70%). Analysis was performed using a random-effects model. The *z*-test result for overall effects showed no statistical significance (*P* = 0.32). We did find significant differences in the mean PedMIDAS score between the two groups (*n* = 205, MD = −9.02, 95% CI = [−17.34, −0.70], *Z* = 2.13, *P* = 0.03) (Fig. 5). The evidence collected in our meta-analysis shows heterogeneity (*I* ^*2*^ = 52%). Analysis was performed suing a random-effects model. The *z*-test result for overall effects was statistically significant (*P* = 0.03).

#### Side effects and adverse events

All included trials reported side effects or adverse events such as paresthesia, weight decrease, anorexia, fever, fatigue, upper respiratory tract infection, somnolence, allergy, and traumatic liver injury. The overall incidence of most adverse events was higher in the topiramate group than in the placebo group, with ten of these events (including suicide attempts and other disabling events) occurring only in the topiramate group. Detailed side effects and adverse events and their frequency in both groups in the included studies are listed in Table 3. We also performed a meta-analysis of each side effect or adverse event that was reported in at least two RCTs﻿. As shown in Fig. 6, there was a significant increase in paresthesia (Fig. 6a, n = 483, RR = 5.04, 95% CI = [2.13, 11.94]; *Z* = 3.68, *P* = 0.0002) and weight decrease (Fig. 6b, n = 380, RR = 4.38, 95% CI = [1.92, 10.01], *Z* = 3.51, *P* = 0.0005) in the topiramate group. The evidence collected in our meta-analysis shows no obvious heterogeneity (*I* ^*2*^ = 0%).Analysis was performed using a fixed-effects model.

**Table 3:** Side effects/Adverse events occurring in any group and ranked by overall incidence

Side effects/Adverse events	Topiramate *N* = 344	Placebo *N* = 139
Paresthesia	71(20.64)^a^	4(2.88)
Upper respiratory tract infection	54(15.7)	16(9.35)
Fatigue	48(13.95)	11(7.91)
Weight decrease	39(11.34)	5(3.59)
Abdominal pain	22(6.40)	12(8.63)
Anorexia	26(7.56)	7(5.03)
Dry mouth	26(7.56)	4(2.88)
Pharyngitis	16(4.65)	13(9.35)
Memory impairment	24(6.98)	3(2.16)
Injury	16(4.65)	11(7.91)
Cognitive disorder	23(6.69)	4(2.88)
Aphasia	23(6.69)	3(2.16)
Sinusitis	14(4.07)	7(5.03)
Nausea	11(3.20)	5(3.59)
Altered mood	14(4.07)	2(1.44)
Dizziness	14(4.07)	1(0.72)
Somnolence	11(3.20)	3(2.16)
Gastroenteritis	10(2.91)	3(2.16)
Fever	10(2.91)	2(1.44)
Influenza-like symptoms	8(2.33)	2(1.44)
Sedation	4(1.16)	2(1.44)
Rhintis	5(1.45)	1(0.72)
Insomnia	4(1.16)	1(0.72)
Viral infection	4(1.16)	1(0.72)
Back pain	2(0.58)	3(2.16)
Conjunctivitis	4(1.16)	1(0.72)
Lack of concentration in school#	4(1.16)	0(0)
Coughing#	4(1.16)	0(0)
Taste perversion#	4(1.16)	0(0)
Abnorma vision	3(0.87)	1(0.72)
Eye pain	2(0.58)	2(1.44)
Vomiting	2(0.58)	1(0.72)
Nervousness^#^	3(0.87)	0(0)
Asthma^#^	2(0.58)	0(0)
Pneumonia^#^	2(0.58)	0(0)
Allergy^#^	2(0.58)	0(0)
Respiratory:bronchospasm	1(0.29)	1(0.72)
Suicide attempt^#^	1(0.29)	0(0)
Intussusception^#^	1(0.29)	0(0)
Traumatic liver injury^#^	1(0.29)	0(0)

An individual subject might have experienced more than one side effect or adverse event

^a^Values expressed as N (%)

#Reported only in the topiramate treatment group

**Fig. 6:**
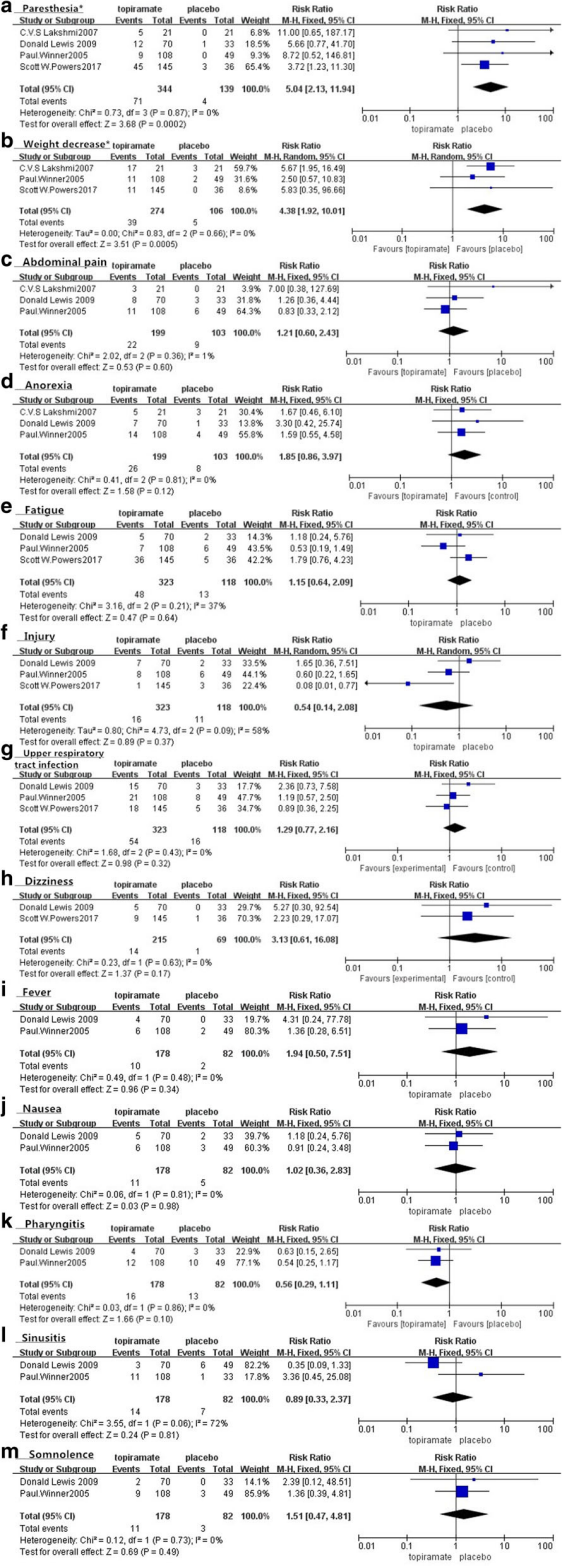
Forest plot of comparison: Side effects/adverse events (a-m, respectively, represent paresthesia,weight decrease, abdominal pain, anorexia, fatigue, injury, upper respiratory tract infection, dizziness, fever, nausea, pharyngitis, sinusitis and somnolence) of topiramate versus placebo(*There was a significant difference between topiramate and placebo groups)

### Publication bias

Since our meta-analysis included only four studies, a linear regression test of funnel plot asymmetry (Egger’s test) could not be performed.

## Discussion

This meta-analysis examined the efficacy of topiramate in comparison with placebo for the prevention of migraines in patients less than 18 years of age. The IHS guidelines for conducting clinical trials indicate that a clinically meaningful end point in a migraine prevention trial is usually defined by a reduction in the total number of headache attacks in a 28-day period or the proportion of patients with a greater than 50% relative reduction in headache frequency [22].

Topiramate is a first-line option for the treatment of migraines in adults, and in March 2014, the U.S. Food and Drug Administration (FDA) approved topiramate for migraine prevention in the population aged 12 to 17 [23]. Moreover, this is the first and only medication currently approved for use in migraine patients 12 years and over. Nevertheless, neither the primary outcome of proportion of patients with a greater than 50% reduction in headache frequency nor the secondary outcome of reduced mean headache days in a 28-day period showed topiramate as more efficacious than placebo in our meta-analysis of four RCTs. According to the definition [22], topiramate showed no statistically significant benefit over placebo in reducing the number of headache days over the treatment period. In fact, the 50% response rate of the topiramate group in 2 trials [14, 20] was not statistically significant compared with the placebo group, and in another trial [18] a similar result was presented for the 50 mg/day topiramate treatment group. The finding conflicts with the outcomes of previous case series and open-label trials. There are at least three possible explanations for this finding. (1) Children tend to have a high placebo response rate, with younger patients in clinical trials demonstrating a greater tendency to respond to placebo. This age-dependent placebo response has ranged from 30% to 70% in migraine studies in general [24–27]. The outcome of our study shows that the average number of patients experiencing a ≥ 50% relative reduction in headache frequency in the placebo group is 50.74% (69/136), which is higher than the rates reported in previous studies of topiramate preventing adult migraine (0–34.2%) [28–31]. Rothner et al. [32] suggested explanations for the higher placebo response rate in clinical trials with children and adolescents, such as the fact that they could not take medication while at school; “good doctor” effects; and the fact that if their symptoms relieved spontaneously, children and adolescents were more likely than adults to believe that they were receiving a drug that had a definite effect on headache. We speculate that this phenomenon is associated with at least the following factors: 1. Different psychological and neurobiological mechanisms exist in pediatric patients compared with adults. There are at least four psychological mechanisms associated with the placebo response: expectation, conditioning, therapeutic relationship and empowerment [33]; psychological mechanisms, especially the conditioning and expectation may guide people’s behavior. The differential course of the maturation of different neurotransmitter systems may explain the differences. 2. The characteristics of migraine attacks are different [34]: migraine headaches in children and adolescents are often bilateral and may be of shorter duration than in adults. 3. Children/adolescents and adults have significantly different cognitive levels. The pain sensation is a highly subjective experience that is influenced by cognitive factors, and placebo analgesia is one of the most striking examples of cognitive regulation of pain [35, 36]. In addition, the lack of pediatric research and the shortage of experience in experimental design may lead to different outcomes. In short, the topic of the difference about placebo response between children/adolescents and adults deserves further discussion. (2) The minimum age at which topiramate was approved for treatment of migraine was 12 years old, but the minimum age of patients in the included trials was 8 years. It is often difficult to calculate the attacks of headache in younger children accurately, and the guardians generally interpret the attacks indirectly from the child’s activity level [19]. (3) Our included patients included those with either episodic or chronic migraine [14], which may influence the results of our meta- analysis.

The second finding of our meta-analysis is that topiramate decreased PedMIDAS scores in migraine patients. PedMIDAS is often used to measure disability related to school absences and functioning, home functioning, and social absences and functioning [16]. This finding, which contradicts our first finding, may indicate that headache-related disability is alleviated by topiramate. However, mean PedMIDAS scores in both the topiramate group and the placebo group decreased between baseline and endpoint, and the fact that only two trials [14, 19] used this tool as a trial assessment may be the cause of the heterogeneity.

As with all antiepileptic drugs, topiramate has many potential side effects or adverse events, some of which may be serious and life-threatening [37]. The rate of adverse events in patients treated with topiramate was higher than that with placebo in our included trials. It has been reported that metabolic acidosis, renal calculi and nervous system effects, such as fatigue or somnolence, paresthesia, dizziness and cognitive disorder or aphasia, occurred in adults and pediatric patients taking topiramate in previous trials. Other adverse events, such as changes in visual acuity, including visual field deficits, acute myopia and secondary closed angle glaucoma, have also been reported. In addition, topiramate (100 mg/day) was related to modest increases in psychomotor reaction times [38]. Another more serious problem is the potential for suicidal behavior and ideation that has been observed in people taking antiepileptic drugs, including topiramate [39]. Thus, while the pathomechanism of migraine is not completely understood, the choice of medication for personalized therapy tailored to each patient needs to be made cautiously [40].

Some limitations in our meta-analysis must be acknowledged. First, because our analysis was limited to articles in the English language literature, we may have omitted some evidence. The secondary limitation is related to the data that we acquired from the four included trials. Three of the trials reported the baseline and follow-up data [14, 18, 19], and one reported baseline and change data [20]. We combined the follow-up and change data according to the research of da Costa, B. R [41]. In addition, one trial compared more than 1 dose [18]; it is likely that dose-finding pharmacologic studies are underrepresented and that additional unpublished industry trials exist. These situations might have resulted in ecological bias. Third, our data had obvious heterogeneity, and none of the variables we abstracted explained this variation. Because we only included four trials and because only three measurements were used in our study, therefore, our findings should be interpreted with caution. The variability in the selection criteria for RCTs and sample size, along with the incomplete reporting of intervention intensity, may also be limitations.

## Conclusions

This is the first meta-analysis of topiramate for migraine prevention in patients less than 18 years of age. We found that topiramate did not achieve a more effective clinical trial endpoint than placebo in the prevention of migraine in patients less than 18 years of age, and topiramate was associated with more adverse events in the included patients. It is possible that a high placebo response rate can be beneficial for children and adolescents with migraine and that drugs used to prevent pediatric migraine might be reconsidered. Because there was a significant placebo response, more placebo-controlled trials in the younger migraine population less than 12 years of age are needed.
